# Advantages and Limitations of Integrated Flagellin Adjuvants for HIV-Based Nanoparticle B-Cell Vaccines

**DOI:** 10.3390/pharmaceutics11050204

**Published:** 2019-05-01

**Authors:** Cornelia Barnowski, Nicole Kadzioch, Dominik Damm, Huimin Yan, Vladimir Temchura

**Affiliations:** 1 Department of Molecular and Medical Virology, Ruhr-University Bochum, 44801 Bochum, Germany; cornelia.barnowski@med.uni-duesseldorf.de (C.B.); nicole.kadzioch@vetsuisse.unibe.ch (N.K.); 2 Institute of Virology, Medical Faculty, Heinrich-Heine-Universität Düsseldorf, 40225 Düsseldorf, Germany; 3 Division of Experimental Clinical Research, Department of Clinical Research and Veterinary Public Health, Vetsuisse Faculty, University of Bern, 3001 Bern, Switzerland; 4 Institute of Clinical and Molecular Virology, Friedrich-Alexander University Erlangen-Nürnberg, 91054 Erlangen, Germany; dominik.damm@uk-erlangen.de; 5 Mucosal Immunity Research Group, State Key Laboratory of Virology, Wuhan Institute of Virology, Chinese Academy of Sciences, Wuhan 430071, China; hmyan@wh.iov.cn

**Keywords:** nano-vaccines, HIV-based VLP, flagellin, B-cell targeting, adjuvant

## Abstract

The great advantage of virus-like particle (VLP) nano-vaccines is their structural identity to wild-type viruses, ensuring that antigen-specific B-cells encounter viral proteins in their natural conformation. “Wild-type” viral nanoparticles can be further genetically or biochemically functionalized with biomolecules (antigens and adjuvants). Flagellin is a potent inducer of innate immunity and it has demonstrated adjuvant effectiveness due to its affinity for toll-like receptor 5 (TLR5). In contrast to most TLR ligands, flagellin is a protein and can induce an immune response against itself. To avoid side-effects, we incorporated a less inflammatory and less immunogenic form of flagellin as an adjuvant into HIV-based nanoparticle B-cell-targeting vaccines that display either the HIV-1 envelope protein (Env) or a model antigen, hen egg lysozyme (HEL). While flagellin significantly enhanced HEL-specific IgG responses, anti-Env antibody responses were suppressed. We demonstrated that flagellin did not activate B-cells directly in vitro, but might compete for CD4+ T-cell help in vivo. Therefore, we hypothesize that in the context of VLP-based B-cell nano-vaccines, flagellin serves as an antigen itself and may outcompete a less immunogenic antigen with its antibody response. In contrast, in combination with a strong immunogen, the adjuvant activity of flagellin may dominate over its immunogenicity.

## 1. Introduction

Virus-like particle (VLP)-based nano-vaccines are a promising tool for HIV-1 vaccine development [1,2,3]. On the one hand, non-infectious VLPs show a higher overall safety profile than life attenuated/inactivated viral vaccines. On the other hand, their structural identity to the prototype viruses provides advantages over synthetically created nano-vaccines in immune system recognition [3,4]. HIV-1-based VLPs can directly target and activate antigen-specific B-cells in vitro [5] and efficiently deliver B- and T-cell antigens into secondary lymphoid organs in vivo [6]. Additionally, VLPs efficiently initiate and modulate B- and T-cell crosstalk both in vitro [7,8] and in vivo [9,10]. Thus, HIV-based VLPs can be considered as efficient B-cell-targeting nano-vaccines.

Flagellin, a principal component of bacterial flagella, is a virulence factor that is recognized by the immune system via the toll-like receptor 5 (TLR5) pathway [11]. An increasing number of studies have demonstrated the effectiveness of flagellin as an adjuvant as well as its ability to promote cytokine production by a range of innate immune cell types. Moreover, it triggers the recruitment of T- and B-cells to secondary lymphoid sites and activates dendritic cells (DCs) and T lymphocytes [12]. Using inorganic nanoparticle B-cell-targeting vaccines functionalized with a model antigen, hen egg lysozyme (HEL), we demonstrated that additional functionalization with flagellin improves antibody responses against HEL in mice [13]. To avoid the high antigenicity of flagellin and the dose-related inflammatory injury induced by flagellin in mice [14,15] we selected a truncated form of nonpathogenic *Escherichia coli* K12 strain-derived flagellin (KF) in which the main antigenicity region (i.e., domains ND2–D3–CD3) was replaced by HIV-1 p24 antigen. The truncated form induced less systemic inflammatory responses and KF-specific antibodies as well as abrogated detectable inflammatory side effects on mice, but kept the adjuvant properties of KF [16]. Here, we generated a membrane-bound form of truncated flagellin (KFΔ) and investigated whether functionalization of HIV-based VLP’s with KFΔ has an adjuvant effect on the immune stimulatory capacities of virus-based nanoparticle B-cell vaccines.

## 2. Materials and Methods

### 2.1. Mice, Ethical Statement

Six- to eight-week-old female C57BL/6J (Bl6) (Janvier, France), Balb/c (Charles River, Germany), and C3H/HeOuJ (C3H) (Charles River, Germany) wild-type (wt) mice, as well as mice with transgenic B-cell receptors (BCR) specific for HIV-1 Env (b12 mice, in-house breeding, kindly provided by Dr. D. Nemazee, The Scripps Research Institute, La Jolla, CA, USA) were used in this study. Mice were housed in singly-ventilated cages in the animal facility of the Faculty of Medicine, Ruhr University Bochum, Germany, in accordance with the national law and were handled according to instructions of the Federation of European Laboratory Animal Science Associations. All animal experiments were approved by an external ethics committee of the North Rhine-Westphalia Ministry for Nature, Environment and Consumer Protection (license 84-02.2011.A111).

### 2.2. Cell Lines, Plasmids

293T cells (obtained from European Collection of Cell Cultures, Salisbury, UK) were cultured in Dulbecco’s modified Eagle Medium (DMEM) (Life Technologies, Carlsbad, CA, USA) with 10% fetal calf serum (FCS) (Life Technologies) and appropriate antibiotics. The plasmids Hgpsyn (a codon-optimized HIV-GagPol sequence) [17], pConBgp140GCD (a codon-optimized HIV-Env clade B consensus sequence) [7], pC–HEL-TM (a sequence of a membrane-anchored form of HEL) [5], pKF (encodes the flagellin sequence of *E. coli* K12 strain MG1655) [18], and pKFD–p24 3D (a sequence of soluble KF in which the domains ND2–D3–CD3 are replaced by HIV p24) [16] have been described.

### 2.3. Construction of an Expression Plasmid Encoding Membrane-Anchored KFΔ (pKFΔ-TM)

**Figure A1:**
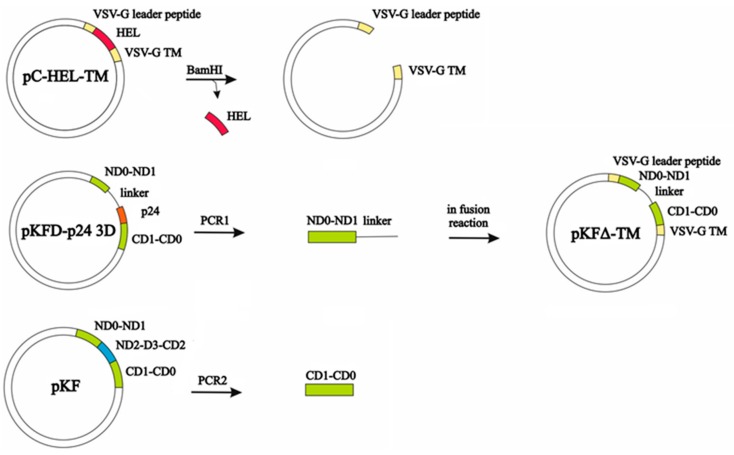
Cloning strategy for the generation of the pKFΔ-TM expression plasmid. The pKF∆-TM expression plasmid consisted of the pC-HEL-TM backbone as well as sequences coding for domains CD1–CD0 of flagellin and the ND0–ND1-linker, which additionally encoded a linker composed of two repeats of 11 amino acids of the human IgG3 hinge region [14]. Because of the VSV-G leader peptide and the VSV-G TM domain, pC-HEL-TM was used as backbone for pKF∆-TM after removal of the HEL sequence by digesting with BamHI. The fragments of the ND0–ND1-linker and CD1–CD0 were separately amplified from the pKFD-p24 3D expression plasmid in PCR1: Primer sequences: ND0–ND1-linker fwd: 5’-CGTGAACTGCGGATCCGCACAAGTCATTAATACCAACAGCCT-3’ and ND0–ND1-linker rev: 5’-ATCCGTGGTTTTACCTCCTGATGTGTGTGTAGTATCACCAAGAGGTGTGC-3’ and from the pKF expression plasmid in PCR2: Primer sequences: CD1–CD0 fwd: 5’-GGTAAAACCACGGATCCGCTGA-3′ and CD1–CD0 rev: 5′-TGTCGCCGAAGGATCCACCCTGCAGCAGAGACAGAACC-3′. The insertion of both fragments into the pC-HEL-TM backbone was performed using the In-Fusion HD Eco Dry Kit from Clontech according to the manufacturer’s protocol.

The pKFΔ-TM expression plasmid was generated by insertion of the amplified fragments ND0–ND1–linker (Linker: With two repeats of 11 amino acids in the human IgG3 hinge region) from plasmid pKFD–p24 3D as well as the sequence CD1–CD0 from plasmid pKF using the In-Fusion HD Eco Dry Kit from Clontech (Figure A1).

### 2.4. VLP Production and Characterization

VLPs were produced as described previously [7] with slight modifications: 293T cells were transiently co-transfected in 175 cm2 flasks using polyethylenimine (PEI) with corresponding plasmids encoding structural and envelope proteins (Table 1). The transfection medium was replaced 6 h after transfection with fresh AIM-V^®^ medium (Life Technologies) and cells were subsequently incubated for 48 h. VLPs were purified and concentrated by ultracentrifugation through a 30% sucrose cushion. The purified VLP pellet was reconstituted in sterile phosphate-buffered saline (PBS), aliquoted, and stored at −80 °C until further use.

Determination of HIV-Gag p24, HIV-Env, and HEL concentrations in VLP preparations was performed with specific ELISAs as reported elsewhere [5,7]. Western blot (WB) analyses for HIV–Gag were performed as reported elsewhere [19]. Rabbit anti-flagellin polyclonal antibodies (ab93713, Abcam) were used for KFΔ protein detection. Secondary anti-rabbit antibodies coupled with horseradish peroxidase (Dako) were used in combination with Chemiglow Kit (Alpha Innotech, San Leandro, CA, USA) to stain the blots. Dynamic light scattering (DLS) was performed with a Zetasizer nanoseries instrument (Malvern Nano-ZS, Malvern Panalytical GmbH, Kassel, Germany). All nanoparticle size data refer to scattering intensity distributions (z-average).

### 2.5. Isolation and Purification of Splenic Cells, In Vitro Culture Experiments

A single-cell suspension of splenic cells was prepared as described before [5]. Naïve untouched B-cells were isolated from single-cell suspension of splenic cells with the B-Cell Isolation Kit (#130-90-862, Miltenyi Biotec, Bergisch Gladbach, Germany). DCs were enriched by positive selection with anti-CD11c magnetic beads (#130-52-001, Miltenyi Biotec). All isolations were performed according to the manufacturer’s instructions. The resulting cells were routinely >98% pure.

Cells in R10 medium (RPMI-1640 (Gibco, Life Technologies, Thermo Fisher Scientific, Carlsbad, CA, USA), supplemented with 10% fetal calf serum, 50 μM β-mercaptoethanol, 10 mM HEPES buffer, and penicillin–streptomycin were plated in U-bottom 96-well plates at a density of 5 × 105 cells in 200 µL R10/well. All VLP preparations were added at the final concentration of 100 ng/mL of HIVEnv. R10 medium serves as a negative control (NIL), and 3 µg/mL of LPS (L6529, Sigma-Aldrich Chemie GmbH, Munich, Germany) were used as a positive control. After 24 h of incubation, the cells were collected, washed, stained with anti-CD11c-APC for DCs, anti-B220-Alexa647 for B-cells, anti-CD86-PeCy7 antibodies (all from eBioscience, Thermo Fisher Scientific), and analyzed by flow cytometry using BD FACSCanto II (BD Biosciences, Heidelberg, Germany) and evaluated with FlowJo (Tree Star, Ashland, OR, USA).

### 2.6. Immunization Experiments, Collection of Blood Samples

Bilateral intramuscular (.) VLP injections were performed in the upper leg under isoflurane anesthesia. Mice received VLPs at the final concentration of 400 ng target antigen per mouse (either HIV-Env for Balb/c mice or HEL for C3H mice). Routinely, mice were immunized twice: At day 0 and at day 35. For serological follow-up, mice were immunized three times: At days 0, 35, and 56.

Mice were bled by puncture of the retroorbital sinus with a heparinized 10 μL hematocrit capillary (Hirschmann Laborgerate, Germany) under isoflurane anesthesia. The sera were obtained after 5 min of centrifugation at 8000 rpm and stored at −20 °C until further use. Routinely, mice were bled at day 0 before immunization (pre-immune sera), at day 28 (3 weeks after the first immunization), and at day 49 (2 weeks after the second immunization). For serological follow-up, additional bleedings were performed at days 63 and 77.

### 2.7. Analyses of Humoral Immune Responses

Antibody responses in 1:100 diluted sera were determined by antigen-specific ELISA as previously described for HIV-Env [7] and HEL [13]. For ELISA measuring of KF-specific antibody responses, recombinant KF protein was produced and purified as described elsewhere [16]. 96 F Maxisorp white microwell plates (Thermo Scientific) were coated with 100 μL of 1 μg/mL of recombinant KF protein in 0.1 M bicarbonate buffer (pH 9.6) overnight at 4 °C. After washing with PBS containing 0.05% Tween-20 (PBS-T), wells were blocked with 5% skimmed milk powder in PBS-T (blocking buffer) for one hour at room temperature (RT). Following another washing step, sera diluted 1:100 in blocking buffer were added to wells for one hour at RT. After washing, the wells were incubated with HRP-conjugated anti-mouse IgG (Bethyl), IgG1, or IgG2a (both from BD Biosciences) diluted 1:1000 in blocking buffer for 1 h at RT. Bound HRP-conjugated antibodies were detected with an enhanced chemiluminescence solution composed of 5 mL Luminol solution (3-aminophtolhydrazide, Sigma-Aldrich), 50 μL solution B (p-coumaric acid, Sigma-Aldrich), and 1.6 μL 30% H2O2 (Merck, Darmstadt, Germany). Humoral immune responses were measured with Berthold Detection Systems Orion Microplate Luminometer (Berthold Technologies) and are expressed as log10-transformed relative light units per second (RLU/s log10).

### 2.8. Characterization of Cellular Immune Responses

Two weeks after the second immunization (day 49), Balb/c mice were sacrificed, draining inguinal lymph nodes were removed, and single-cell suspensions were prepared. Cells from non-immunized animals served as a negative control (contr.). Cells were re-stimulated with MHC-II-restricted HIV-Env peptide GVPVWKEATTTLFCASDAKA in the presence of 2 μg/mL anti-CD28 (37.51; eBioscience) and 2 μmol monensin as described elsewhere [9]. After 6 h of stimulation, cells were surface-stained with anti-CD4-FITC and intracellular-stained with anti-IFN-γ-PE antibodies (all from eBioscience) as described [9]. Data were acquired on BD FACSCanto II (BD Biosciences) and analyzed with FlowJo (Tree Star).

### 2.9. Statistical Analysis

Calculations were performed as indicated in the figure legends using GraphPad Prism 7 software (GraphPad, San Diego, CA, USA).

## 3. Results and Discussion

### 3.1. Generation of a Membrane-Bound Form of Truncated KF for HIV-VLP Functionalization

HIV-derived enveloped VLPs can efficiently serve as surface antigen displays consisting of a common HIV-Gag protein backbone and the cellular lipid membrane in which the antigen of interest can be expressed. Previously, we functionalized HIV-Gag viral nanoparticles with model antigens that originally are soluble non-viral proteins [5].

**Figure 1:**
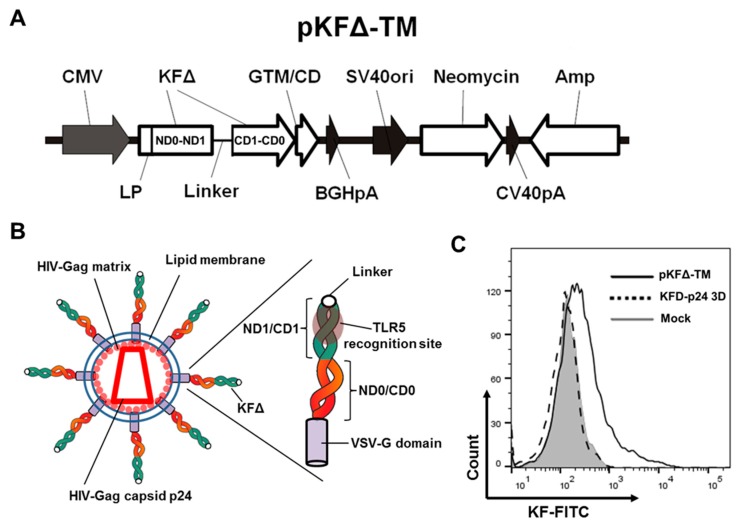

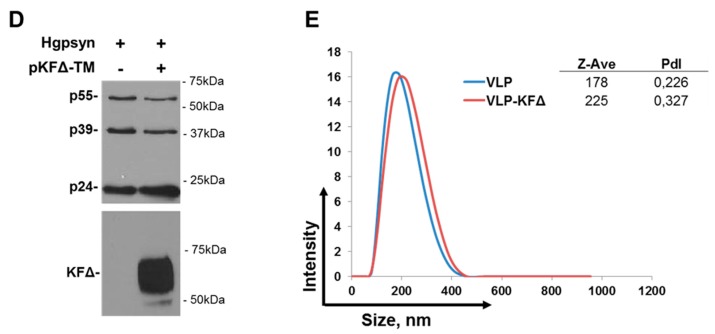
Membrane-anchored form of KFΔ for HIV-VLP functionalization. (**A**) Schematic representation of the pKFΔ-TM plasmid. Amp: Ampicillin resistance; BGHpA: Bovine growth hormone polyadenylation signal; CD: Cellular domain of VSV-G; CMV: Cytomegalovirus promoter; GTM: Transmembrane domain of VSV-G; LP: Leader peptide of VSV-G; SV40ori: Simian virus 40 promoter; SV40pA: Simian virus 40 polyadenylation signal. (**B**) Diagram of HIV-based VLP with membrane-anchored KFΔ (VLP-KFΔ) and single membrane-anchored KFΔ domain. (**C**) FACS analysis of 293T cells stained for the presence of KF protein on the cell surface two days after transfection with the expression plasmid pKFD-p24 3D or pKFΔ-TM encoding a soluble or a membrane-anchored KFΔ protein, respectively. (**D**) 293T cells were co-transfected with Hgpsyn alone or together with pKFΔ-TM plasmid. Pelleted nanoparticles obtained by ultracentrifugation of the conditioned media were analyzed for the presence of KF and HIV-Gag proteins by Western blot analyses. (**E**) Dynamic light scattering data of VLP and VLP-KFΔ particles with average particle diameter in nm (Z-ave) and polydispersity index (PdI).

Now, we used this genetic approach to integrate a truncated form of soluble bacterial flagellin of a nonpathogenic *E. coli* strain that lacks the domains D2 and D3 into HIV-Gag-based viral nanoparticles. Deletion of hypervariable domains ND2–D3–CD3 reduced the immunogenicity of the protein and the systemic inflammatory response against it, but retained the TLR5 agonist activity [16,20]. We used sequences of original plasmids [5,16] (Figure 1A) to insert N-terminal D0–D1 domains (ND0–ND1) connected via a linker with C-terminal D1–D0 domains (CD1–CD0) between the sequences coding for the leader peptide and the transmembrane, as well as the cytoplasmic domains of the vesicular stomatitis virus G-protein (VSV-G). Figure 1A represents the resulting pKFΔ-TM construct. The rationale behind this design was: (i) To create HIV-based nanoparticles displaying multiple flagellin molecules on the surface with an orientation optimized for TLR5 recognition [21], and (ii) to enhance molecule flexibility and achieve cis-dimerization of the N-terminal and C-terminal D0–D1 domains by introducing a flexible linker in-between (Figure 1B).

The flow cytometry analysis of HEK293T cells transiently transfected with pKFΔ-TM or with soluble KFD-p24 3D confirmed the presence of KFΔ protein on the cell surface in the expected orientation as a type 1 membrane protein exposing its N-terminus to the extracellular space (Figure 1C). After co-transfection of 293T cells with pKFΔ-TM and Hgpsyn (a codon-optimized expression plasmid that encodes HIV-1 GagPol proteins), VLPs could be pelleted by ultracentrifugation through a sucrose cushion. Western blot analyses demonstrated the presence of both HIV-Gag (p55, Gag; p24, capsid) and KFΔ in the pelleted nanoparticles (Figure 1D). HIV-Gag-mediated budding does not change the orientation of the protein, and therefore KFΔ is presented at the outer surface of the nanoparticles as an ordinary envelope protein.

As reported by Yuan Lu et al., when D0-stabilized flagellin was chemically attached to non-enveloped Hepatitis B core protein VLPs with the D0 domain facing outward, the tendency of flagellin to polymerize caused the VLPs to precipitate. However, attaching the D0 domain to the VLP surface produced a stable nanoparticle adjuvant [21]. According to our pKFΔ-TM design, KFΔ flagellin domains have capacities for cis-dimerization during the particle production by the cell, and released nanoparticles have D0 domains facing the particle surface (Figure 1A,B). To control trans-dimerization and nanoparticle conjugation, we measured HIV-Gag protein backbone VLPs and VLPs functionalized with KFΔ (VLP-KFΔ) by dynamic light scattering. No abnormal VLP-KFΔ precipitates were observed (Figure 1E).

Thus, we developed an effective genetic method for functionalization of HIV-based viral nanoparticle membranes with the truncated form of flagellin.

### 3.2. Activation of Antigen-Presenting Cells in Vitro

To analyze the immunomodulatory effects of KFΔ functionalization on the activation of antigen-specific (cognate) and non-specific (non-cognate) B-cells, we produced native HIV-1 nanoparticles, containing GagPol and Env proteins of HIV-1 (Env-VLP), and Env-VLPs functionalized with KFΔ (Env-VLP- KFΔ). The amount of Env (the antigen of interest) in VLP preparations was routinely measured with Env ELISA, and the presence of KFΔ was confirmed with WB.

**Figure 2:**
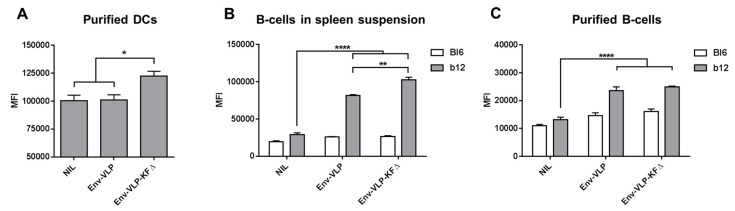
Activation of dendritic cells (DCs) and cognate B-cells with Env-VLP-KFΔ nano-vaccines. (**A**) DC, (**B**) splenocyte, or (**C**) B-cell suspensions from wild-type (wt) Bl6 or BCR-transgenic b12 mice were incubated for 24 h in the presence of same amounts of either Env-VLP or Env-VLP-KFΔ. After incubation, the cells were stained with anti-CD11c (**A**), anti-B220 (**B**,**C**), and anti-CD86 (**A**–**C**) antibodies and analyzed with flow cytometry. Data are depicted as mean fluorescent intensity (MFI) of anti-CD86 antibody staining for CD11c^+^ DC cells (**A**) and B220^+^ B-cells (**B**,**C**). Data are presented as the means ± SEM of three independent experiments. * *p* < 0.05; ** *p* < 0.005; **** *p* < 0.0001; one-way ANOVA with Tukey multiple comparison post-hoc test.

As it was shown before, soluble KFD-p24 3D activates DCs from wt mice, but not from TLR5-knockout mice in vitro [14]. Therefore, we first proved the bioactivity of the KFΔ-functionalized nanoparticles on freshly isolated splenic DCs from wt Bl6 mice. DCs were cultured in the presence of either Env-VLP or Env-VLP-KFΔ. The results clearly indicate the bioactivity effects of the KFΔ-functionalized nanoparticles on the primary innate immune cells (Figure 2A).

Previously, we demonstrated that HIV-based nanoparticle vaccines can directly target and activate naive cognate B-cells in vitro [5]. To investigate whether KFΔ functionalization is able to: (i) further improve the activation capacities of Env-VLP on cognate HIV-Env-specific B-cells, and (ii) induce polyclonal activation of non-cognate B-cells, we incubated spleen cell suspensions from wild-type Bl6 and BCR-transgenic b12 mice with either Env-VLP or Env-VLP-KFΔ. LPS stimulation was used as an internal positive control for the cell activation inducibility (all cells used could be activated with LPS, data not shown). B-cells from b12 mice are able to recognize HIV-Env with their BCRs, which results in B-cell activation. Functionalization of Env-VLPs with KFΔ significantly increased the activation capacities of the Env-based nanoparticles on the cognate B-cells, but had no influence on non-cognate Bl6 B-cells (Figure 2B).

The observed adjuvantive effect on cognate B-cells might be either due to: (i) a synergistic mode of BCR activation and the direct sensing of KFΔ by activated B-cells, or (ii) a synergistic effect of BCR activation and paracrine influence of KFΔ-activated innate immune cells from the spleen suspension. To evaluate the nature of the adjuvantive effect (direct vs. indirect), we isolated primary b12 B-cells out of the spleen cell suspensions and directly stimulated them with Env-VLP or Env-VLP-KFΔ. In contrast to the spleen cell suspensions, no adjuvantive effect of KFΔ functionalization was observed (Figure 2C). These data are consistent with results of Gururjan et al., who demonstrated that mouse naive follicular B-cells do not express TLR5 and do not respond to flagellin stimulation in vitro [22]. Taken together, HIV-1-based viral nanoparticles demonstrated a direct stimulatory effect on dendritic cells and an indirect adjuvantive effect on VLP-stimulated cognate B-cells in co-culture systems after functionalization with KFΔ.

### 3.3. Modulation of Env-Specific Antibody and CD4+ T-Cell Responses In Vivo

**Figure 3:**
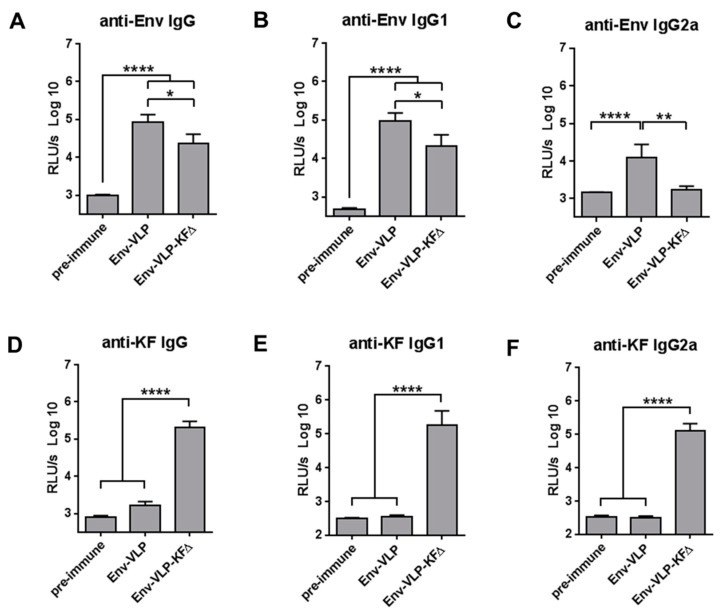

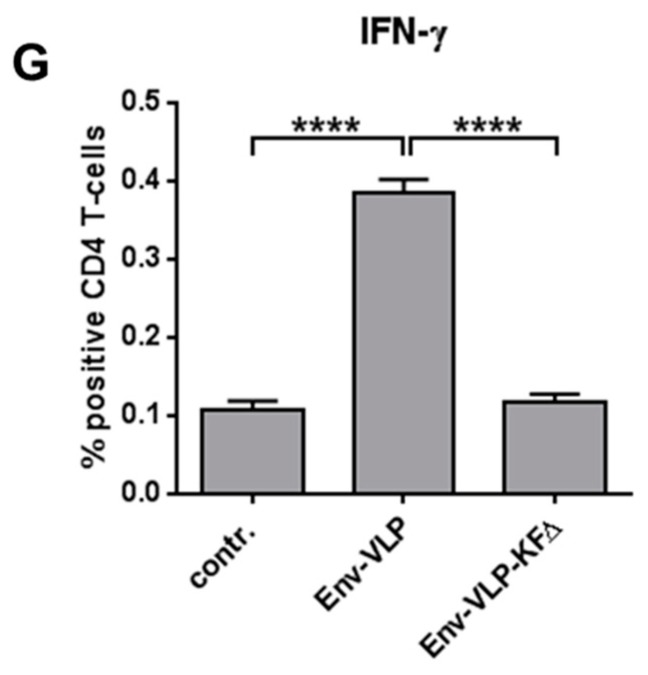
Immunogenicity of Env-VLP nano-vaccines functionalized with KFΔ. *Wt* Balb/c mice were immunized i.m. twice (at day 0 and day 35) with either Env-VLP or Env-VLP-KFΔ containing 400 ng HIV-Env/mouse. The sera samples were obtained at day 0 before immunization (pre-immune sera) and at day 49 (2 weeks after the second immunization). Humoral immune responses against HIV-Env (**A**–**C**) and KF (**D**–**F**) were measured in 1:100 diluted sera samples and expressed as log10-transformed relative light units per second (RLU/s log10). Each experimental group included six mice. The pre-immune sera group contains samples of 12 mice correspondingly. The columns represent the mean values ± SEM. (**G**) Characterization of HIV-Env-specific cellular immune responses in the draining lymph nodes was performed at day 49 (2 weeks after the second immunization). Percentage of CD4+ T-cells producing IFN-γ after in vitro stimulation with HIV-Env T helper peptide was measured by intracellular cytokine staining. The columns represent the mean values of six animals ± SEM. * *p* < 0.05; ** *p* = 0.0015; **** *p* < 0.0001; one-way ANOVA with Tukey multiple comparison post-hoc test.

In order to analyze the adjuvantive effects of KFΔ functionalization on the induction of anti-Env-specific antibody responses, we immunized wt Balb/c mice with Env-VLP or Env-VLP-KFΔ containing the same amounts of HIV-Env antigen. Nanoparticles were applied intramuscularly (a clinically relevant administration route) two times over the four-week interval. Two weeks after the second immunization, total anti-Env IgG antibody responses (Figure 3A) as well as anti-Env-specific IgG1 (Figure 3B) and IgG2a (Figure 3C) subclasses in sera of immunized mice were measured and compared with those in sera before immunizations (pre-immune).

**Figure A2:**
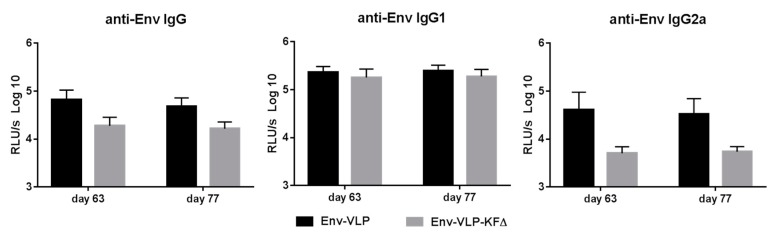
Immunogenicity of Env-VLP nano-vaccines functionalized with KFΔ (serological follow up). For serological follow up, *Wt* Balb/c mice were immunized i.m. three times: at days 0, 35, and 56 with either Env-VLP or Env-VLP-KFΔ containing 400 ng HIV-Env/mouse. The sera samples were obtained at days 63 and 77. Humoral immune responses against HIV-Env were measured in 1:100 diluted sera samples and expressed as log10-transformed relative light units per second (RLU/s log10). The columns represent the mean values ± SEM.

Unpredictably, the KFΔ functionalization significantly decreased anti-Env Ab levels of total IgG and IgG1 subclass (Figure 3A,B) levels and totally abrogated induction of IgG2a (Figure 3C), the predominant antiviral IgG antibody subclass in the mouse [23]. To exclude that the KFΔ functionalization only transiently decreases antibody responses, mice were immunized three times and a serological follow-up over 11 weeks was performed. Anti-HIV-Env-specific IgG antibody levels induced by Env-VLP-KFΔ immunization continued to be lower than in Env-VLP immunized mice (Figure A2). Then, we measured the immunogenicity of KFΔ and observed strong induction of anti-KF-specific antibodies with equally strong production of both IgG1 and IgG2a antibodies (Figure 3D–F).

In the splenocyte co-cultures, we observed the adjuvantive effect of KFΔ on Env-specific B-cell activation (Figure 2B). However, in vivo compartmentalization of the immune system components might prevent such indirect effects. After i.m. HIV-based VLP injection, DCs take up the nanoparticles, become activated, and migrate into the T-zones of the secondary lymphoid organs to further activate and instruct cognate CD4+ T-cells. Simultaneously, a direct contact with VLPs facilitates B-cell activation in B-cell zones [6,24]. Activated cognate T- and B-cells migrate to contact each other, because the T-/B-cell collaboration is essential for the generation of antibody-producing plasma cells [24].

Since (i) primary in vivo activation of antigen-specific B-cells requires a direct cognate VLP triggering rather than interactions with VLP-loaded DCs [6] and (ii) Env-VLP-KFΔ demonstrated no direct adjuvantive effect on Env-specific B-cells (Figure 2C), in vivo induction of cognate CD4+ T-cells by DCs might play a role in the regulation of anti-Env antibody production [25,26].

We analyzed Env-specific CD4+ T-cell responses after immunization with Env-VLP vs. Env-VLP-KFΔ. IFN-γ production by CD4+ T-cells after re-stimulation with HIV-Env MHC-class-II restricted immunodominant peptide was significantly impaired in mice immunized with Env-VLP-KFΔ (Figure 3G), which is consistent with the strong decline in IgG2a antibodies [27].

Taken together, immunization of Balb/c mice with HIV-1-based viral nanoparticles functionalized with KFΔ decreased the Env-specific CD4+ T-cell activation and anti-Env antibody production, while anti-KF antibodies were prominently produced.

### 3.4. Adjuvantive Effect on HEL-Specific Antibody Responses In Vivo

The results obtained after Env-VLP-KFΔ immunization of Balb/c mice, however, were in contradiction with data previously published by us [13] and others [28]. Using B-cell targeting calcium–phosphate (CaP) nanoparticles functionalized with hen egg lysozyme as a model antigen, we demonstrated that additional functionalization with the full length flagellin from *Salmonella enterica* significantly improved anti-HEL antibody responses in C3H mice [13].

**Figure 4:**
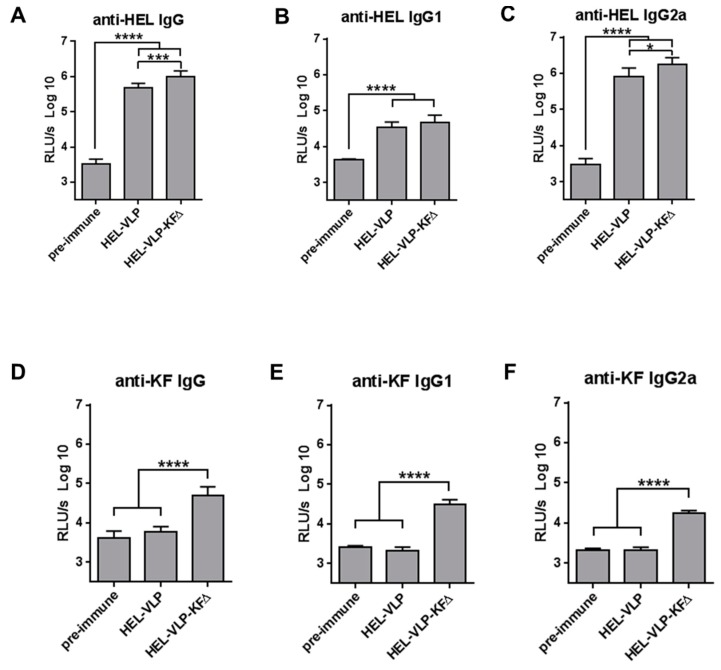
Induction of humoral immune responses in C3H mice with HEL-VLP nano-vaccines functionalized with KFΔ. *Wt* C3H mice were immunized i.m. twice (at day 0 and day 35) with either HEL-VLP or HEL-VLP-KFΔ containing 400 ng of HEL/mouse. The sera samples were obtained at day 0 before immunization (pre-immune sera) and at day 49 (2 weeks after the second immunization). Humoral immune responses against HEL (**A**–**C**) and KF (**D**–**F**) were measured in 1:100 diluted sera samples and expressed as log10-transformed relative light units per second (RLU/s log10). Each experimental group included six mice. the pre-immune sera group contains samples of 12 mice correspondingly. The columns represent the mean values ± SEM. * *p* = 0.0169; *** *p* = 0.0009; **** *p* < 0.0001; one-way ANOVA with Tukey multiple comparison post-hoc test.

To verify these results for KFΔ functionalization in the context of HIV-based viral nanoparticles, we produced HEL-VLP (VLPs carrying HEL as a surface antigen [5]) and HEL-VLP functionalized with KFΔ (HEL-VLP-KFΔ) (see Table 1). After immunization of C3H mice with either HEL-VLP or HEL-VLP-KFΔ, we observed positive adjuvantive effects of KFΔ functionalization on the induced anti-HEL antibody responses (Figure 4A–C). The anti-HEL total IgG (Figure 4A) and IgG2a (Figure 4C) subclass levels were significantly higher in mice immunized with HEL-VLP-KFΔ, while induced anti-KF antibody responses remained relatively moderate (Figure 4D–F vs. Figure 3D–F). Thus, functionalization of B-cell-targeting HEL-VLP vaccines with KFΔ demonstrated an adjuvantive effect and increased anti-HEL IgG antibody responses in C3H mice.

### 3.5. Immunogenicity Balances between the Target Antigens and KFΔ

**Figure 5:**
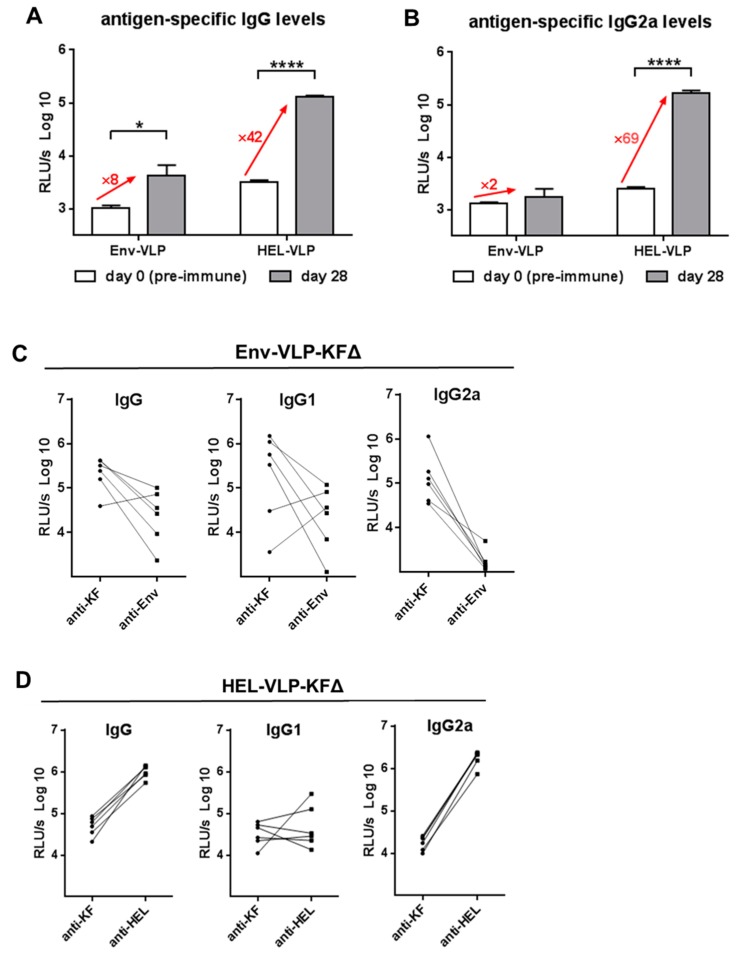
Immunogenicity balance between different envelope proteins of HIV-based nano-vaccines. (**A**,**B**) Wt mice were immunized with Env-VLP (Balb/c) or HEL-VLP (C3H) in total amount of 400 ng antigen/mouse. 28 days after the single immunization total IgG (**A**) and IgG2a (**B**) humoral responses against corresponding antigens were measured in 1:100 diluted sera samples and expressed as log10-transformed relative light units per second (RLU/s log10). The columns represent the mean values ± SEM (*n* = 6). * *p* = 0.0329; **** *p* < 0.0001; two-tailed paired *t*-test. Fold of increase in the signal intensity (numbers in red) was calculated for each individual mouse (*n* = 6) as ratio: (Untransformed signal at day 28)/(untransformed signal at day 0). Means of six values are present. (**C**,**D**) Data represent humoral immune responses against KF vs. HIV-Env (**C**) and KF vs. HEL (**D**) in the individual Balb/c (**C**) and C3H (**D**) mice at day 49 (2 weeks after the second immunization with Env-VLP-KFΔ (**C**) or with HEL-VLP-KFΔ (**D**)).

C3H mice are genetically predisposed to strongly react to HEL protein with antibody production [29]. We compared induction of the total IgG humoral immune response (Figure 5A) and the potent antiviral IgG2a subtype response (Figure 5B) in C3H mice against HEL proteins with those in Balb/c mice against HIV-Env proteins four weeks after a single immunization with corresponding VLPs. Based on the differences in the magnitude of antigen-specific antibody induction on the same amount of antigens between Balb/c and C3H mice, one might consider that HIV-Env is a rather weak immunogen in Balb/c mice.

Although at least one Env MHC-class-II-restricted immunodominant peptide for Balb/c mice is known, after immunization with Env-VLP-KFΔ, there was no induction of Env-specific IFN-γ producing CD4+ T-cells observed. This lack of T-cells was accomplished with diminished anti-Env IgG2a antibody responses. At the same time, significant levels of anti-KF IgG2a antibodies were detected, suggesting that KFΔ has no negative influence on IgG2a class-switch per se (Figure 3). One can speculate that in the context of VLP-based B-cell nano-vaccines, KFΔ serves as a protein antigen itself and may outcompete a less immunogenic antigen with its antibody response via the CD4+ T-cell-dependent mechanism, which was recently described as a model of B-cell competition for T-cell help [30]. In contrast, in combination with a strong immunogen (such as HEL in C3H mice) the adjuvant activity of flagellin may dominate over its immunogenicity. This might explain the contradictory data previously published by Vassilieva et al., in which flagellin functionalization enhanced the Env-specific humoral responses to Env-VLP immunization in guinea pigs [28]. Guinea pigs are phylogenetically distant from mice and have higher immunoglobulin combinatorial diversity [31]. These animals are broadly used in HIV studies due to their ability to induce HIV-Env neutralizing antibodies [32], which might indicate a stronger immunogenicity of HIV-Env in guinea pigs than in mice.

To consolidate our hypothesis, we analyzed the immunogenicity balances between the VLP envelope antigens and the KFΔ adjuvant for individual Balb/c and C3H mice. The results summarized in Figure 5C–D provide an empirical support of the antigen competition idea and suggest that by functionalization of viral nanoparticle vaccines, the immunogenicity of flagellin itself has to be taken into consideration. The adjuvantive potency of flagellin might be dependent on both the immunogenicity of each particular antigen used and the genetic background (e.g., MHC-class-II variants) of the vaccine.

In contrast to MHC-class-II mediated presentation to CD4+ T-cells, cross-presentation of VLP-derived antigens to CD8+ T-cells is usually restricted to a subset of CD8+ DCs [33]. However, this CD8+ DC subset that has the ability to cross-present [34] demonstrates the lowest level of relative TLR5 expression in comparison to the other conventional DCs [35]. This implies the need for comprehensive studies on the cross-presentation of flagellin-functionalized VLPs by different DC subsets. Although induction of cytotoxic T-lymphocyte (CTL) responses for prophylactic HIV-1 vaccines was formally concluded in 2007 with the unexpected lack of efficacy in the STEP trial [36], the genetic functionalization of cell-derived nanoparticles (enveloped VLPs and exosomes [37]) with truncated flagellin might be of interest for the further development of CTL-inducing anti-viral and anti-cancer vaccines.

## 4. Conclusions

In summary, we developed an effective genetic method for functionalization of HIV-1-based viral nanoparticle membranes with a truncated form of flagellin. KFΔ is presented at the outer surface of the nanoparticles as an ordinary envelope protein. Rational design of the KFΔ molecule prevents precipitation of nanoparticles and exposes the TLR-5 binding site outwards. KFΔ-functionalized HIV-based nanoparticles demonstrated bioactivity in vitro. Functionalization of B-cell-targeting viral nanoparticle vaccines with KFΔ demonstrated both inhibitory and adjuvantive immunostimulatory effects on humoral immune responses against target antigens. The overall outcome of immunizations was based on the immunogenicity balance between the antigen and KFΔ. Therefore, in contrast to other TLR-ligands, the antigenic property of flagellin in comparison to the immunogenicity of the target antigen has to be considered for the functionalization of viral nanoparticle vaccines.

## Figures and Tables

**Table 1:** HIV-based virus-like particle preparations used in the study.

Abbreviations	Envelope Proteins	Structural Proteins
VLP	-	HIV-Gag/Pol ^7^
VLP-KFΔ	KFΔ ^1^	HIV-Gag/Pol ^7^
Env-VLP	HIV-Env ^2^	HIV-Gag/Pol ^7^
Env-VLP-KFΔ	HIV-Env ^3^; KFΔ ^4^	HIV-Gag/Pol ^7^
HEL-VLP	HEL ^5^	HIV-Gag/Pol ^7^
HEL-VLP-KFΔ	HEL ^6^; KFΔ ^4^	HIV-Gag/Pol ^7^

^1^ Plasmid pKFΔ-TM, 40 μg per transfection; ^2^ plasmid pConBgp140GCD, 40 μg per transfection; ^3^ plasmid pConBgp140GCD, 20 μg per transfection; ^4^ plasmid pKFΔ-TM, 20 μg per transfection; ^5^ plasmid pC-HEL-TM, 40 μg per transfection; ^6^ plasmid pC-HEL-TM, 20 μg per transfection; ^7^ plasmid Hgpsyn, 40 μg per transfection.
